# Behavioral Improvement and Regulation of Molecules Related to Neuroplasticity in Ischemic Rat Spinal Cord Treated with PEDF

**DOI:** 10.1155/2014/451639

**Published:** 2014-07-03

**Authors:** Chary Marquez Batista, Leonardo Luis Torres Bianqui, Bruno Bonganha Zanon, Mauricio Menezes Aben Athar Ivo, Gabriela Pintar de Oliveira, Jessica Ruivo Maximino, Gerson Chadi

**Affiliations:** Neuroregeneration Center, Department of Neurology, School of Medicine, University of São Paulo, Avenida Dr. Arnaldo 455, 2nd Floor, Room 2119, 01246-903-São Paulo, SP, Brazil

## Abstract

Pigment epithelium derived factor (PEDF) exerts trophic actions to motoneurons and modulates nonneuronal restorative events, but its effects on neuroplasticity responses after spinal cord (SC) injury are unknown. Rats received a low thoracic SC photothrombotic ischemia and local injection of PEDF and were evaluated behaviorally six weeks later. PEDF actions were detailed in SC ventral horn (motor) in the levels of the lumbar central pattern generator (CPG), far from the injury site. Molecules related to neuroplasticity (MAP-2), those that are able to modulate such event, for instance, neurotrophic factors (NT-3, GDNF, BDNF, and FGF-2), chondroitin sulfate proteoglycans (CSPG), and those associated with angiogenesis and antiapoptosis (laminin and Bcl-2) and Eph (receptor)/ephrin system were evaluated at cellular or molecular levels. PEDF injection improved motor behavioral performance and increased MAP-2 levels and dendritic processes in the region of lumbar CPG. Treatment also elevated GDNF and decreased NT-3, laminin, and CSPG. Injury elevated EphA4 and ephrin-B1 levels, and PEDF treatment increased ephrin A2 and ephrins B1, B2, and B3. Eph receptors and ephrins were found in specific populations of neurons and astrocytes. PEDF treatment to SC injury triggered neuroplasticity in lumbar CPG and regulation of neurotrophic factors, extracellular matrix molecules, and ephrins.

## 1. Introduction

The effects of exogenous administration of neurotrophic molecules on neurorestoration of lesioned central nervous system have been largely evaluated. Pigment epithelium derived factor (PEDF) has emerged as a potential candidate to promote behavioral gain and neurorestorative events in the spinal cord injury because PEDF receptor is concentrated in ventral motor region of the spinal cord and the molecule is highly expressed and it is able to trigger trophic actions to motor neurons [1, 2]. The potential capacity of PEDF to promote motor restoration may involve its ability to modulate neurotrophic/neuroplasticity events and its capacity to interfere with endothelial and glial reactions to injury, cells that are also important actors in the scenario of neurorestoration [2–5].

Despite the great effort to understand the inhibitory nature of neuronal environment to regenerating fibers, recent results have pointed out neuronal plasticity in the lesioned spinal cord as the major event leading functional recovery in that pathological condition [6–10]. In fact, studies have identified and characterized molecular signals related to axonal growth and functional target innervation in the lesioned spinal cord. Much of the functional restoration observed in the experimental models of spinal cord lesion has been triggered however by the neuroplasticity-related molecules that are expressed by rescued neurons nearby injury, especially in the regions of the central pattern generators (CPG) [11–14]. Furthermore, the apoptotic and neurotrophic responses in neurons that are not enrolled by primary lesion as well as the paracrine glial mechanisms may modify the functional outcome [15–17]. Those events occurred in several rostral/caudal spinal levels in the subsequent periods after lesion [18, 19].

Molecular signaling plays a central role in the cell communication-mediated neuroplasticity in the lesioned nervous system, especially those that are involved in neuroprotection, neovascularization, glial activation, and extracellular matrix regulating neuronal fiber growth. In that context, neurotrophin 3 (NT-3), glial derived neurotrophic factor (GDNF), brain derived neurotrophic factor (BDNF), fibroblast growth factor 2 (FGF-2), and PEDF have been described as proteins that are able to exert autocrine/paracrine trophic actions to spinal cord neurons and also able to modulate wound repair events that are triggered by nonneuronal cells [5, 16, 20]. Furthermore, the ephrin system may also play important action on neurorestoration, remarkably in the lesioned spinal cord, due to its actions on multiple cellular regenerative events. For instance, ephrins could modulate axonal guidance [21–23], target reinnervation, synaptic plasticity [24–26], neurotrophic factor signaling-induced neuroprotection/neuroplasticity, and endothelial/glial reaction-induced wound repair [27–29]. The large range of actions of ephrin system is favored by the spread distribution of the receptor tyrosine kinase Eph and its membrane-bound ligand ephrin in astrocytes, endothelial cells as well as in presynaptic and postsynaptic neurons [30–32].

Remarkably, the above mentioned intercellular signaling events that mediate the outcome of a spinal cord injury are modulated by molecules of the extracellular matrix specially the members of the chondroitin sulfate proteoglycan (CSPG) family. Two molecules, which were revealed by immunoblot analysis after chondroitinase treatment, are in the context of neuroregeneration. The 70 kDa chondroitin sulfate/dermatan sulfate hybrid chain [33] and also the 150 kDa neurocan-derived C-terminal product, a proteolytic and biological activity fraction of the full-length neurocan [34, 35], seem to play a role in neurotrophic factor binding and neuronal fiber growth.

Among the vast, complex, and imbricate cellular and molecular events driving neurorestorative functional outcome, the local ischemia is a pathological condition that appears not only as a consequence of lesion reaction to a spinal cord injury but also as a disorder that is able to interfere with local wound repair and neurotrophic/neuroplasticity mechanisms [36, 37]. The effects of a PEDF injection in the epicenter of an ischemic lesion applied in a low thoracic level of the rat spinal cord were investigated behaviorally and also at cellular and molecular levels. Regulations of molecules related to neuroplasticity, neurotrophism, and neovascularization and also those of the growth cone inhibitor chondroitin sulfate proteoglycans (CSPG) family and ephrin system were analyzed in the lumbar spinal cord CPG.

## 2. Materials and Methods

### 2.1. Photothrombotic Spinal Cord Injury

Specific pathogen-free adult male Wistar rats from the University of São Paulo School of Medicine (*n* = 42, weighting 350–400 g) were used in the present study. Rats were kept under controlled temperature and humidity conditions with a standardized light/dark cycle (light on at 7 : 00 a.m. and off at 7 : 00 p.m.) with free access to food pellet and tap water. The study was conducted according to protocols approved by the Animal Care and Use Ethic Committee at the University of São Paulo and in accordance with the Guide for the Care and Use of Laboratory Animals adopted by the National Institutes of Health.

Rats were anesthetized intraperitoneally with a mixture of ketamine chlorhydrate (62.5 mg/kg) and xylazine chlorhydrate (10 mg/kg). The dorsal vein of the rat penis was exposed. Rose Bengal dye (Aldrich Chemical, 40 mg/kg, diluted in 0.9% NaCl in a final concentration of 20 mg/mL) was administered slowly in the rat dorsal vein (*n* = 30). The rat back was shaved and disinfected with an iodopovidone solution. Immediately after dye injection, a midline longitudinal incision was made over the thoracic 8- to the lumbar L1 (T8-L1) vertebrae; the skin was retracted and paravertebral muscles were dissected. The spinal process and the vertebral lamina of thoracic 12 (T12) spinal cord level were removed with a complete exhibition of circular regional dura mater (laminectomy). A 5 mm diameter-fiber-optic linked to a xenon lamp device (Schott KL 1500, Mainz, Germany), which produces a 560 nm wavelength irradiation, was placed on the surface of the dura-exposed spinal cord (thoracic level) illuminating it for 20 minutes. The fiber-optic was attached to a stereotaxic device (Kopf, USA), attempting to maintain a 2 mm distance between the fiber-optic and the dorsal spinal cord to be lesioned. The procedure induced an excitation of the systemically injected dye, triggering a local thrombosis and ischemia [38, 39]. Sham operated rats (*n* = 12) were submitted to microsurgical procedures and light exposure, without receiving the dye intravenous injection.

### 2.2. Injection of PEDF

Immediately after spinal cord ischemia or sham surgery, the animals received a stereotaxical injection of the neurotrophic factor PEDF (10 *μ*L, 100 ng/mL; HS300210, kindly supplied by Dr. Joyce Tombran-Tink, University of Missouri-Kansas City) or solvent (10 *μ*L, phosphate buffered saline, pH 7.4) at the epicenter of the lesion. Animals were kept anesthetized with halothane inhalation during the period of spinal cord injections. The spinal injections were made with 100 *μ*m outer diameter glass needles, which were obtained by means of a pipette puller (Kopf). The needle was connected to a Hamilton syringe (50 *μ*L) and to a stereotaxical apparatus adapted with a spinal cord unit (Kopf) in order to promote the mechanical injection, which lasted 3–5 minutes. Three experimental groups were performed. In the Sham group rats were submitted to a sham surgery and a local injection of solvent (*n* = 12). In the Saline group rats received an ischemic lesion and a local injection of solvent (*n* = 14). In the PEDF group rats received an ischemic lesion and a local injection of PEDF (*n* = 16).

At the end of the procedure, the muscular layer and skin were sutured. The animals received preventive antibiotic therapy (ceftriaxone, 40 mg/kg, im. daily) for 15 days and also accompanied for bladder evacuation using the Crede method until bladder functional recovery.

### 2.3. Behavioral Analysis

Animals submitted to photothrombotic spinal cord injury or sham operation were evaluated behaviorally using the inclined plane test 24, 48, and 72 hours after surgery and then weekly until the end of week 6. Animals were placed head down on an adjustable inclined plane covered by a rubber mat. The angle of the plane was increased from 0° to the point where the rat could not maintain its position for 5 seconds. Two measurements were performed for each rat. Normal uninjured rats remained on the plane to an angle of 50 to 60° inclination [40]. The inclined plane test was chosen because the performance on the inclined plane correlates with the integrity of the rubrospinal tract and other nonpyramidal pathways [41] that could be compromised at week 6 after ischemic injury. The test can be also used as an index of animal strength and it has been shown to be a sensitive and reliable test for clip compression injury [42].

### 2.4. Immunohistochemical Procedures

Half of the animals in each group (*n* = 6–8) were processed for immunohistochemistry six weeks after spinal cord injury. The rats were anesthetized with sodium pentobarbital and euthanized by a transcardiac perfusion with 100 mL isotonic saline at room temperature, followed by 500 mL of fixation fluid (4°C) over a period of 6 minutes [20, 43, 44]. The fixative consisted of 4% paraformaldehyde (w/v, Merck, Darmstadt, Germany) in 0.1 M phosphate buffer, pH 6.9. The spinal cords were removed, kept in the fixative solution at 4°C for 90 minutes, and then rinsed in 10% sucrose (Merck) dissolved in 0.1 M phosphate-buffered saline (PBS), pH 7.4, for 48 hours. The spinal cords were cut into pieces of 0.8 cm long at the lumbar intumescence level. The segments were then frozen in dry ice-cooled (−40°C) isopentane (Sigma, Milwaukee, WI) and stored at a −70°C freezer until use.

#### 2.4.1. Sectioning and Sampling

Adjacent serial 14 *μ*m thick frozen sections were obtained with a cryostat (Leica, CM3000, Germany) from the spinal cord lumbar segment (L2–L5), containing the CPG region [45]. Series in a cranial-caudal order including every 100th sections were used for tissue labeling. Thus, three sampling sections/animal were submitted to each immunolabeling procedure (see below).

#### 2.4.2. One-Color Immunoperoxidase

Immunoreactivity was detected by the avidin-biotin peroxidase technique [46, 47]. Sections were washed for 2 × 10 minutes in PBS and incubated with 5% normal goat serum for 30 minutes at room temperature. The series of sections were then incubated for 48 hours at 4°C with the mouse monoclonal microtubule associated protein-2 antibody (MAP-2, diluted 1 : 2,500, Sigma). The antibody was diluted in PBS containing 0.5% Triton X-100 (Sigma) and 1% bovine serum albumin (Sigma). The sections were washed again in PBS (2 × 10 minutes) and incubated with biotinylated horse anti-mouse immunoglobulins (Vector, Burlingame, CA, USA; diluted 1 : 250) for 2 hours. After rinsing in PBS, sections were incubated with an avidin-biotin peroxidase complex (both diluted 1 : 125, Vectastain, Vector, USA) for 45 minutes. Immunoreactivity was visualized using 3-3′-diaminobenzidine tetrahydrochloride (Sigma) as a chromogen and H_2_O_2_ (0.05%, v/v, Sigma) for 8 minutes. To standardize the immunohistochemical procedure, we used a dilution of the primary antibody, a DAB concentration, and an incubation time, all adjusted so that the darkest elements in the spinal cord sections were below saturation. To further analyze the specificity of the immunostainings, sections were incubated with the solvent of the primary and secondary antibodies or with the solvent of the avidin-biotin solution and processed at the same time with the experimental sections. The sections submitted for measurements were selected from those three of sampling regime described above that represented better the cranial (L2-L3) and caudal (L4-L5) regions of the lumbar rat spinal cord. The means of the data of the two regions were presented because there is no description of functional differences between two regions of the CPG.

#### 2.4.3. Image Analysis

The MAP-2 immunoreactivity was measured in two spinal cord sections per rat by means of semiquantitative morphometric image analysis. The measurements were done in the ventral horn of lumbar intumescence (caudal to the injury site), where the lumbar CPG is located and the spinal cord showed a preserved morphology. The image analysis procedures, implemented on a Kontron-Zeiss KS400 image analyzer (Germany), have been described previously [19, 43, 46, 48–50]. Briefly, a television camera from the microscope (×40 objective) acquired the image. The fields were selected within the ventral horn of the gray matter, bilaterally. After shading correction, a discrimination procedure was performed according to the following protocol. The mean gray value (MGV) and s.e.m. of gray matter in areas of the spinal cord devoid of specific labeling (background, bg) were measured. Gray values darker than bgMGV, 3 s.e.m. were considered as belonging to specific labeling and thus discriminated. The specific (sp) MGV was subsequently defined as the difference between the bgMGV value and the MGV of discriminated profiles. The glass value was left constant at 200 MGV. The procedure was repeated for each section to correct every measurement of specific labeling for its background value. The morphometric and microdensitometric (spMGV) measurements indicate the amount of immunoreactive cell profiles and the intensity of immunolabeling in the sampled fields, respectively.

#### 2.4.4. Stereological Analysis

The MAP-2 immunoreactive dendritic and cell body profiles were quantified individually by means of a stereological method. The point intercepts stereological tool was employed to obtain the areal fraction [29] in the sampled region of the ventral horn of the spinal cord rats, bilaterally, as described elsewhere [20, 43, 44]. A point-grid with an area per point of 400 *μ*m^2^ was used to estimate the area of the counted profiles. The points hitting all immunoreactive profiles (∑*P*_structure_) were counted. The points hitting the section (∑*P*_section_) were also counted. The AA was calculated [20, 43, 44] (AA = ∑*P*_structure_/∑*P*_section_). A 40x oil-immersion objective was used to acquire the images that were used to quantify the profiles in the gray matter sampled fields. All MAP-2 immunoreactive processes that emerged from the cell perikarya (first level processes) were not considered in the discrimination procedures so that axons and axon hillock were not included in the measurements.

#### 2.4.5. Two-Color Immunofluorescence

This method was employed for simultaneous detection of immunoreactivities. The sections were initially washed for 3 × 10 minutes in PBS and then were incubated for 48 hours at 4°C with a mixture of one of the antibodies to receptor Eph or ephrin subtypes (the descriptions of the antibodies and the employed dilutions are shown in Table 1) and one of the following antibodies to neuronal and astroglial markers: mouse monoclonal neurofilament-200 antibody (NF-200, diluted 1 : 200, Sigma), rabbit polyclonal glial fibrillary acidic protein antibody (GFAP, diluted 1 : 200, Sigma), or goat polyclonal GFAP antibody (diluted 1 : 50, Dako). The analysis allowed the cellular localization of Eph receptors and ephrins in those cells. The antibodies were diluted in PBS containing 0.5% Triton X-100 (Sigma) and 1% bovine serum albumin (Sigma). After the incubation of the primary antibodies, sections received two washes of 10 minutes in PBS and were incubated for 2 hours in the dark at 37°C with a mixture of fluorescein isothiocyanate (FITC) and Texas Red (both diluted 1 : 40, Jackson, West Grove, USA). The sections were rinsed in PBS and were examined in an AX70 Olympus epifluorescence photomicroscope.

### 2.5. Western Blot and Real-Time PCR Techniques

The other half of the animals (*n* = 6–8) were euthanatized by decapitation and processed to western blot and real-time polymerase chain reaction (RT-PCR) techniques. The spinal cords were rapidly removed and a 0.8 cm fragment was cut from the lumbar intumescence as described previously. The ventral part (motor area) was carefully separated from the dorsal part (sensory area) in each fragment. The ventral region was then subdivided into two parts. The caudal portion was then submitted to western blot technique and the cranial one to the RT-PCR procedures. The material of RT-PCR was frozen in dry ice and stored at −70°C freezer until processing.

#### 2.5.1. Western Blot

The samples were homogenized in lyses buffer containing 1% NP40 (Sigma), 0.5% sodium deoxycholate (Sigma), 1% sodium dodecyl sulfate (Biorad), 1 mM ethylenediaminetetraacetic acid (Sigma), 1 mM ethylene glycol tetraacetic acid (Sigma), and 1% protease inhibitor cocktail (Sigma), diluted in phosphate buffered (pH 7.4). After centrifugation (14,000 rpm) for 20 min at 4°C, as described previously [51], the supernatants were transferred into new tubes and stored at −70°C until use.

Protein concentrations were determined according to the method described by Bradford. The samples (60 *μ*g of protein/lane) were separated on a 12% sodium dodecyl sulfate (SDS) polyacrylamide (BioRad) gel electrophoresis at 100 V for 1 h. Proteins (120 *μ*g) were transferred to polyvinylidenefluoride (PVDF) membrane at 100 V during one hour.

Membranes were then blocked with 10% milk diluted in TBS-T (mixture of tris-buffered saline and 0.05% Tween 20) for 30 minutes under slight agitation at room temperature. Then, all membranes were incubated overnight at 4°C with the respective antibodies: a mouse monoclonal antibody to MAP-2 (1 : 1,000; Novus Biological), a mouse monoclonal antibody to brain core protein of CSPG (1 : 500, Millipore), a rabbit polyclonal antibody to laminin (1 : 1,000; Sigma), and a rabbit polyclonal antibody to B-cell lymphoma protein-2 (Bcl-2, diluted 1 : 1,000; Santa Cruz). Membranes were also submitted to specific labeling of subtypes A and B of Eph receptors and ephrins. The concentration used and the descriptions of the antibodies are shown in Table 1. The antibodies were diluted in 3% milk/TBS-T. Membranes were washed 2 times for 10 minutes in TBS-T and incubated at room temperature for 1 hour with anti-mouse (1 : 6,000; GE), anti-rabbit (1 : 10,000; GE), or anti-goat (1 : 2,000; GE) IgG–ECL conjugated secondary antibodies. In the sequence, membranes were washed two times with TBS-T and once in TBS. After final washes, the membranes were incubated with Western Lightning Chemiluminescence Reagent Plus (PerkinElmer Life Science, USA) for 1 minute. The membranes were exposed to an X-ray film for imaging (HyperfilmTM ECL, GE healthcare, USA) to visualize protein bands. The membranes of every blot were then washed and incubated individually with a mouse monoclonal antibody to alpha-tubulin (1 : 30,000; Sigma) diluted in TBS-T containing 1% BSA for 1 hour at room temperature and developed as described previously. Thus, all blot signals were accompanied for their respective alpha-tubulin labeling. The densitometry of the bands was quantified by means of a computer assisted image analyzer and a software developed by Imaging Research (Brock University, Canada) as described elsewhere [51]. Dividing the density of the protein signal by the correspondent alpha-tubulin signal value performed data normalization.

#### 2.5.2. RNA Extraction and Real-Time PCR

Total RNA from spinal cord fragments was extracted with RNAspin Mini Kit (GE Healthcare, UK) according to the manufacturer's instructions. RNA quantity and integrity were assessed by spectrophotometry (Nanodrop) and microfluidics-based electrophoresis (Bioanalyzer, Agilent 2100), respectively. Only RNA samples with OD 260/280 ≥ 1.8 and RIN (RNA integrity number) >7 were used for quantitative RT-PCR.

For RT-PCR reactions, total RNA was converted into cDNA in the presence of reverse transcriptase (Multiscribe, Applied Biosystems) and random hexamer primers (Applied Biosystems), according to manufacturers' instructions. Quantitative PCRs reactions were performed on a Step One Plus sequence detection system (Applied Biosystems) using Taqman Universal PCR Master Mix (Applied Biosystems) in a total volume of 20 *μ*L. No-template reactions were used as negative controls.

The gene expression of the following molecules was analyzed: the neurotrophic factors NT-3 (Rn00579280_m1), GDNF (Rn00569510_m1), BDNF (customized, Forward 5′TGGTTATTTCATACTTCGG TTGCATGA3′, Reverse 5′TGTCCGTGGACGTTTGCTT3′, and Probe 5′CTGCGCCCATGAAAG3′), and FGF-2 (Rn00570809_m1), the Eph receptors EphA6 (Rn01474859_m1) and EphB2 (Rn01181017_m1), and the small GTPase RhoA (Rn04219610_g1). Data were normalized with 18S (Applied Biosystems). The amplification protocol included 3 minutes at 95°C, followed by 45 cycles of 10 seconds at 95°C for denaturation and 45 seconds at 60°C for annealing and extension.

### 2.6. Statistical Analysis

Statistical analysis for the behavioral data consisted of a two-way analysis of variance [22] and followed the Bonferroni posttest. A second analysis was performed including only the Saline and PEDF groups, in order to obtain data related to motor recovery of the injured animals. In the western blot, immunohistochemistry and RT-PCR analyses, the one-way ANOVA was applied with Tukey's multiple comparisons posttest to identify statistical significances between groups. All analyses were performed using Prism 5.0 (Graph Pad, CA). Data were presented as means ± s.e.m. and significance level was set at *P* < 0.05.

## 3. Results

### 3.1. Behavioral Analysis

**Figure 1:**
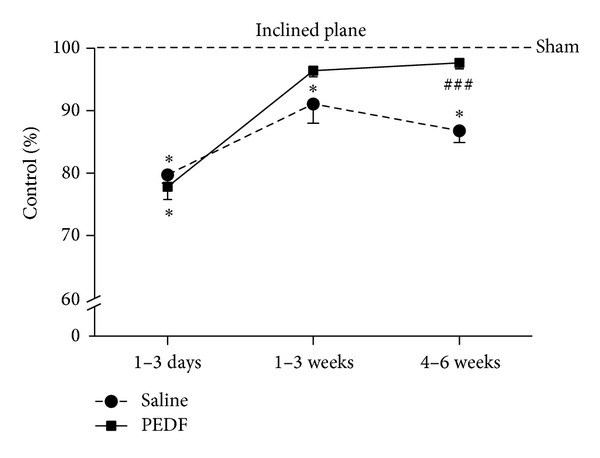
Inclined plane test was employed to evaluate motor behavior recovery or rats that received a sham operation (Sham) or a photothrombotic injury at the low thoracic level of the spinal cord and were treated with a local injection of phosphate buffered saline (Saline) or pigmented epithelial derived factor (PEDF). Values were expressed as a percentage of the Sham group. The tests were performed daily until the third day and then weekly until the sixth week after surgery and were grouped into three periods (1–3 days; 1–3 weeks; 4–6 weeks). Two-way ANOVA showed differences on motor recovery of animals regarding time of post injury, treatment, and the interaction between them, as described in the text. Bonferroni posttest pointed out differences between groups [*(*P* < 0.05) when Saline or PEDF groups differ from Sham; ^###^(*P* < 0.001)] when the PEDF group differs from Saline. Means ± s.e.m., *n* = 6–8.

The motor evaluation of animals submitted to ischemic spinal cord injury was performed by the inclined plane test. The statistical analysis by the two-way ANOVA showed effects of time (*P* < 0.0001; *F* = 101.4), treatment (*P* < 0.0001; *F* = 32.99), and interaction time/treatment (*P* < 0.0001; *F* = 7.516) when the three groups were analyzed together, as well as when only the Saline and PEDF groups were included in the evaluation (effects of time *P* < 0.0001, *F* = 99.14; of treatment *P* = 0.002, *F* = 9.543; and of interaction time/treatment *P* = 0.004, *F* = 5.80). Therefore, differences between the Saline and PEDF groups (*P* < 0.001) were seen at week 4 to week 6 of assessment, according to the Bonferroni posttest (Figure 1). Furthermore, although Sham and Saline groups differed at all evaluated periods, Sham and PEDF groups were different only at 1–3 days time-interval (Figure 1).

### 3.2. Analysis of Neuroplasticity

**Figure 2:**
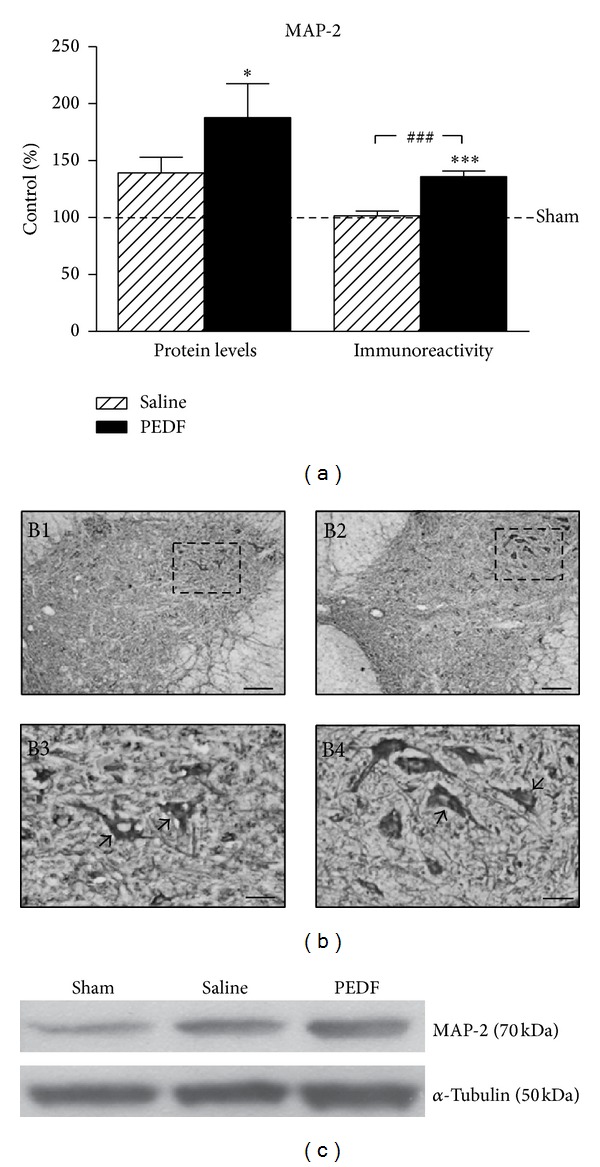
Levels (a) of microtubule associated protein 2 (MAP-2) in the ventral horn of the lumbar spinal cord of Sham, Saline, and PEDF groups 6 weeks after surgery, detected by western blot (Protein Levels) and microdensitometric/morphometric image analysis (Immunoreactivity). Rats received a sham operation (Sham) or a photothrombotic injury at the low thoracic level of the spinal cord and were treated with a local injection of phosphate buffered saline (Saline) or pigmented epithelial derived factor (PEDF). Means ± s.e.m., *n* = 6–8. Values were expressed as a percentage of the Sham group. *(*P* < 0.05) and ***(*P* < 0.001) are differences of PEDF group compared to Sham, and ^###^(*P* < 0.001) is the difference of PEDF group compared to Saline according to one-way ANOVA followed by Tukey posttest. Microphotographs illustrated MAP-2 immunoreactivity (b) of rats submitted to a sham surgery (B1, B3) or a photothrombotic injury followed by PEDF (B2, B4). The microphotographs B3 and B4 are higher magnifications of regions depicted from B1 and B2, respectively. The bands of the MAP-2 and alpha (*α*) Tubulin signals (c) by means of western blot are also illustrated. Scale bars: 100 *μ*m (B1, B2) and 30 *μ*m (B3, B4).

**Figure 3:**
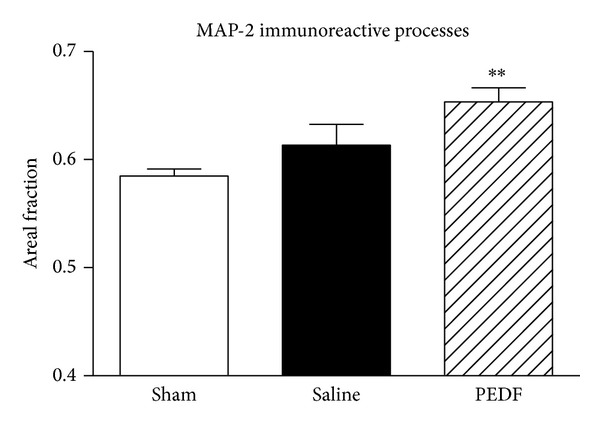
Measurements of the microtubule associated protein 2 (MAP-2) immunoreactive neuronal processes (dendritic processes) in the ventral horn of the lumbar spinal cord of the rats, bilaterally, by means of stereological tool point intercepts. The values were expressed as Area Fraction. See text for details. The rats of the Sham, Saline, and PEDF groups were sacrificed six weeks after surgery. The amount of MAP-2 dendritic profiles was increased in the lesioned rats that received a PEDF spinal cord injection. PEDF group differed from Sham group (**P < 0.01), according the one-way ANOVA followed by Turkey posttest. Means ± s.e.m., *n* = 6–8.

The effects of photothrombotic ischemic injury and treatment with PEDF on the neuroplasticity in the ventral horn of the lumbar spinal cord were analyzed by means of MAP-2 detection. Western blot revealed an increase (87.78%) of MAP-2 protein level in the ventral region of lumbar spinal cord of PEDF treated group compared to Sham (*P* < 0.05, Figure 2(a), also illustrated in Figure 2(c)). Moreover, MAP-2 immunoreactivity was elevated (*P* < 0.001) in the ventral horn of lumbar spinal cord in rats of PEDF group compared with the Sham (35.99%) and Saline (33.81%) groups (Figure 2(a)). The qualitative analysis showed an increased MAP-2 immunoreactivity in the neuronal cell bodies and fibers of the spinal cord ventral horn of PEDF rats, compared to Saline rats (Figure 2(b)). Furthermore, the stereological tool point intercepts revealed that PEDF injection elevated (11.71%, values expressed as Areal Fraction) the amount of MAP-2 immunoreactive dendritic processes in the ventral horn of the lumbar spinal cord in relation of Saline injected rats (Figure 3). The stereological method revealed no alterations in the Areal Fraction of the MAP-2 immunoreactive perikarya among experimental groups. The values of Areal Fraction of MAP-2 immunoreactive perikarya were 31.69 ± 1.61, 34.79 ± 2.17, and 38.94 ± 4.39 of Sham, Saline, and PEDF groups, respectively (mean ± s.e.m., *P* > 0.05).

**Figure 4:**
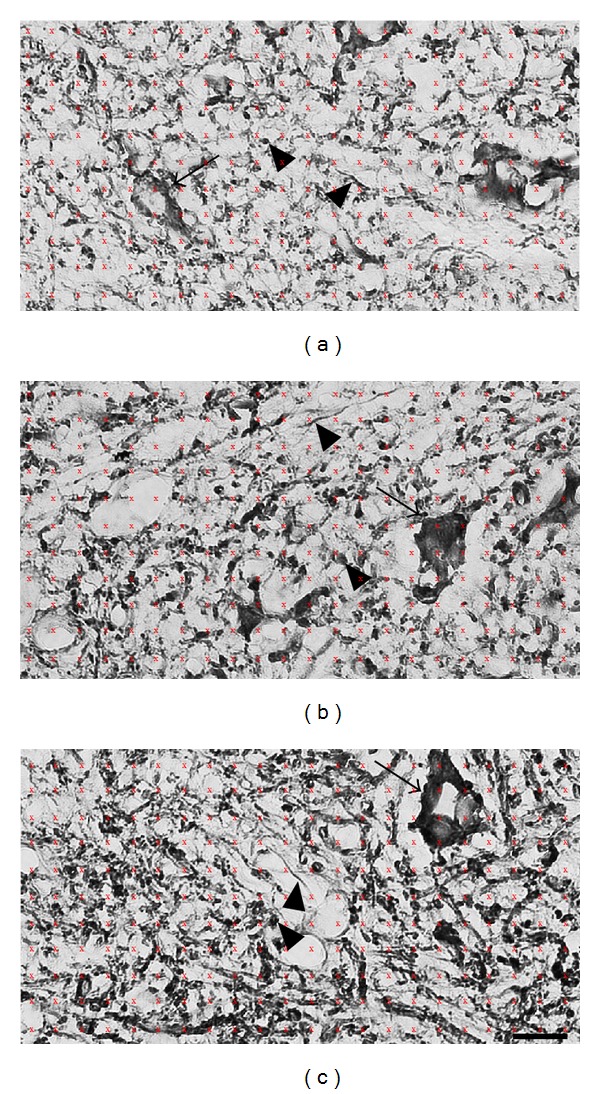
High magnification microphotographs illustrate MAP-2 immunoreactivity in the ventral horn of the lumbar spinal cord of Sham (a), Saline (b), and PEDF (c) groups of rats. Animals were sacrificed six weeks after surgery. The stereological tool point intercepts (reddish color frame of an area per point of 400 *μ*m^2^) were employed in the quantifications of MAP-2 positive dendrites and cell bodies of the rat spinal cord ventral horn bilaterally. Scale bars: 40 *μ*m.

The increased MAP-2 immunoreactive dendritic profiles in the ventral horn of PEDF treated rats are illustrated in higher magnification microphotographs of representative rats of the three studied groups (Figure 4). The stereological tool, named point intercepts (red frame), that was projected on the digital image of MAP-2 immunoreactive profiles for quantification of neuronal dendrites and cell bodies of ventral horn were illustrated in Figure 4.

It should be mentioned that the sections incubated with the solvent of the primary or secondary antisera as well as with the solvent of the avidin-biotin solution showed no reaction (data not shown).

### 3.3. Analysis of Neurotrophic Factors

RT-PCR for relative gene expression of neurotrophic factors in the ventral region of lumbar spinal cord revealed a decrease of NT-3 in the PEDF group compared to Sham (38.52%, *P* < 0.05, Table 2). Moreover, GDNF gene expression was found to be elevated in the PEDF group compared to Sham (38.21%, *P* < 0.05) and also to Saline (94.0%, *P* < 0.01) groups (Table 2). No differences were seen in the analyses of BDNF and FGF-2 gene expression (Table 2).

PEDF protein level was also quantified in the studied region by western blot. No differences were observed among the experimental groups at the postoperative periods performed in this study (data not shown), thus representing no autocrine regulation of that neurotrophic factor in the present experimental conditions.

### 3.4. Analysis of Growth Inhibitory Molecule

**Figure 5:**
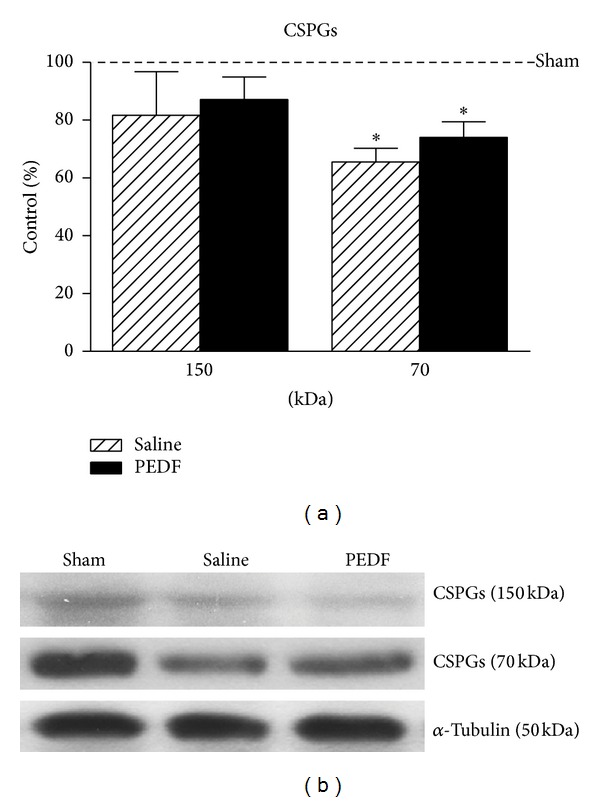
Levels (a) of chondroitin sulfate proteoglycan (CSPG, 150 and 70 kDa molecular weight forms; see text for details), detected by western blot, in the ventral region of the lumbar spinal cord of Sham, Saline, and PEDF groups 6 weeks after surgery. Values of the relative optical density of the signal in the bands were expressed as a percentage of the Sham group. Mean ± s.e.m., *n* = 6–8. The representative bands are illustrated in (b). *α*-tubulin (50 kDa) was used as sample loading control. *(*P* < 0.05) when the Saline or PEDF groups differ from Sham, according the one-way ANOVA followed by Tukey posttest.

Western blot of the growth cone inhibitor CSPG levels in the ventral region of lumbar spinal cord showed two bands of molecular weights 70 and 150 kDa (Figures 5(a) and 5(b)). The chondroitinase treatment allowed the identification of a 70 kDa chondroitin sulfate/dermatan sulfate hybrid chain [33] and also a 150 kDa neurocan-derived C-terminal product [34, 35]. Decreases in the CSPG level were found in the 70 kDa band (*P* < 0.05) of the PEDF (12.92%) and also Saline (18.33%) groups compared to Sham rats (Figure 5(a), also illustrated in Figure 5(b)).

### 3.5. Biochemical Analyses of Angiogenesis and Apoptosis

**Figure 6:**
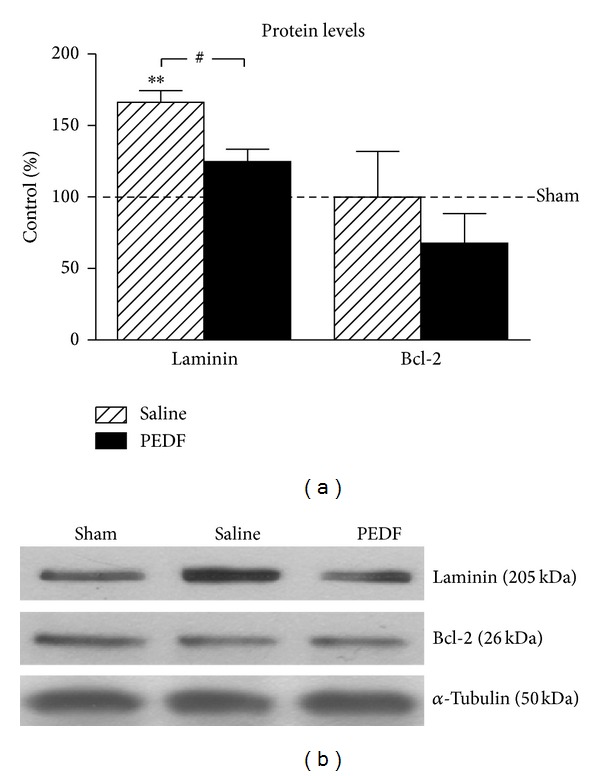
Levels of laminin (205 kDa) and B-cell lymphoma 2 antiapoptotic factor (Bcl-2, 26 kDa), detected by western blot, in the ventral region of the lumbar spinal cord of Sham, Saline and, PEDF groups 6 weeks after surgery. Values of the relative optical density of the signal in the bands were expressed as a percentage of respective Sham groups. The representative bands are illustrated in (b), and *α*-tubulin (50 kDa) was used as sample loading control. **(*P* < 0.01) when the Saline group differs from Sham and ^#^(*P* < 0.05) when the PEDF group differs from Saline group, according the one-way ANOVA followed by Tukey posttest. Means ± s.e.m., *n* = 6–8.

Laminin and Bcl-2 proteins were used as markers for angiogenesis and antiapoptosis regulator, respectively, and were quantified by means of western blot in the ventral region of lumbar spinal cord of the rats (Figures 6(a) and 6(b)). Laminin level was elevated (66.23%) in the Saline group compared to Sham (*P* < 0.01) and a decrease (24.97%) in the level of laminin was found in the PEDF group compared to Saline (*P* < 0.5). Regarding the Bcl-2 analysis, no changes were demonstrated among groups at the postoperative studied periods (Figure 6).

### 3.6. Analysis of Eph/Ephrin System

**Figure 7:**
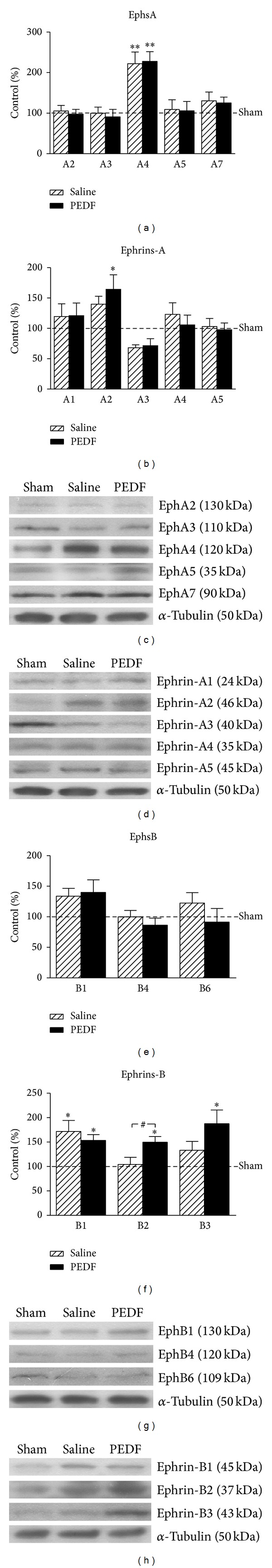
Levels of type A Eph receptors (A2–5 and A7, (a)), type A ephrins (A1–5, (b)), type B Eph receptors (B1, B4, and B6, (e)), and type B ephrins (B1–3, (f)), detected by western blot, in the ventral region of the lumbar spinal cord of Sham, Saline, and PEDF groups 6 weeks after surgery. Values of the relative optical density of the signal in the bands were expressed as a percentage of respective Sham groups. The representative bands of type A Eph receptors and ephrins are illustrated in (c) and (d), respectively, and the representative bands of type B Eph receptors and ephrins are illustrated in (g) and (h), respectively. *α*-tubulin (50 kDa) was used as sample loading control. *(*P* < 0.05) and **(*P* < 0.01) when the Saline/PEDF group differs from Sham and ^#^(*P* < 0.05) when the PEDF group differs from Saline, according the one-way ANOVA followed by Tukey posttest. Means ± s.e.m., *n* = 6–8.

The protein levels of subtypes A and B of the Eph receptors and ephrins were quantified by western blot in the ventral region of lumbar spinal cord of the rats (Figure 7). The EphA4 level increased in the Saline (122.16%) and PEDF (127.93%) groups compared to Sham (*P* < 0.01), and no changes were observed in protein levels of the receptors EphA2, A3, A5, and A7 (Figure 7(a)). Regarding the levels of type-A ephrins, ephrin A2 increased (64.28%) in the PEDF group compared to Sham (*P* < 0.05), and no changes were seen in the ephrins A1, A3, A4, and A5 (Figure 7(b)). The representative bands of Ephs A and ephrins A illustrated the effect described above (Figures 7(c) and 7(d)).

Furthermore, regarding the Ephs B1, B4, and B6 (Figure 7(e)), no differences were found among the experimental groups. Moreover, increases of ephrin B1 levels were observed in the Saline (71.78%) and PEDF (53.51%) groups compared to Sham (*P* < 0.05, Figure 7(f)). Ephrin B2 level was elevated in the PEDF group compared to Sham (49.67%) and Saline (43.54%) groups (*P* < 0.05), and the ephrin B3 level was increased in the PEDF group (87.55%) compared to Sham (*P* < 0.05), (Figure 7(f)). The representative bands of Ephs B and Ephrins B illustrated the effects described above (Figures 7(g) and 7(h)).

Some members of studied Eph receptors, particularly the EphA1, A6, A8, A10, B2, and B3, showed no specific signal bands at the western blot assay. Finally, the relative gene expression analyses of EphA6 and EphB2 receptors as well as the small GTPase RhoA, performed by RT-PCR in an adjacent region, showed no differences among the experimental groups (Table 2).

### 3.7. Analysis of Eph/Ephrin Cellular Localization

**Figure 8:**
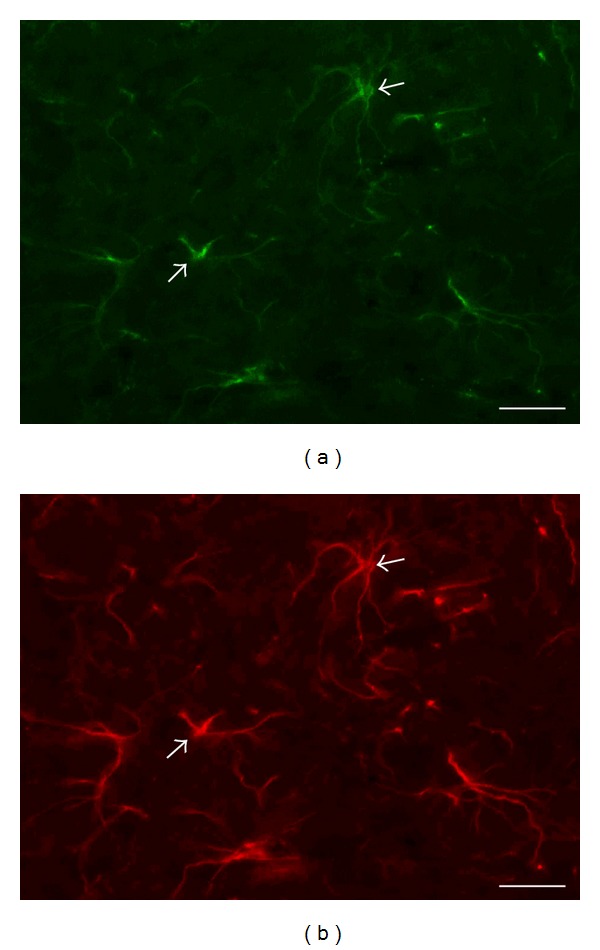
Photomicrographs of two-color immunofluorescence procedures showing the cellular localization (arrows) of ephrin B2 ((a), green) in glial fibrillary acidic protein (GFAP) positive astrocytes ((b), red) in the ventral horn of lumbar spinal cord of rat that received a low thoracic photothrombotic injury and PEDF injection. Scale bar = 20 *μ*m.

The cellular analysis of the two-color immunofluorescence in ventral horn of the rat spinal cord showed a colocalization of the EphA1, EphA2, EphA7, EphA8, EphB1, EphB4, and EphB6 receptors, as well as the ephrin A5 with the NF-200 positive motor neurons of this region. The type B ephrins (B1, B2, and B3) are colocalized with astrocytes of that region (data not shown). The presence of ephrin B2 in astrocytes of ventral horn of PEDF treated rat is illustrated in Figure 8. The qualitative analysis of immunofluorescence allowed the recognition of cellular component of the labeling signals but did not lead the identification of differences among groups.

## 4. Discussion

### 4.1. Spinal Cord Injury Model of Photothrombotic Ischemia

Spinal cord ischemia is considered the most relevant event present in a series of pathological situations, including trauma, tumor, infection, and circulatory disorders, contributing to the deleterious secondary mechanisms that amplify initial injury [52–55]. Ischemia might also influence injury outcome and neurorestorative events due to its ability to modify neurotrophic and neuroplasticity responses of nearby lesion [56, 57].

Experimental models of spinal cord trauma are largely employed [58–62]; however, analyses have failed to attempt the circumstances of related ischemia. This work performed a spinal cord injury photothrombotic ischemia using the Rose Bengal method, as first described by Watson et al. [63], that has been widely employed in the cerebral ischemia studies. Reproducible lesion with regard to its size, localization, and behavioral response is achieved because the method allows a precise control of dye concentration and irradiation settings thus leading, in rats, to a nonrecovery of blood flow [64], an absence of short term behavioral recovery [65], and a 3-month-old circumscribed lesion in the dorsal half of the cord as described previously [66–68]. Importantly, the photothrombotic injury applied at the low thoracic levels of rat spinal cord of the present work preserved the lumbar segments caudally where rat lumbar CPG is located [69].

Neuroplasticity in the lumbar CPG has been addressed as the major source of motor recovery, spontaneously or after treatments, following spinal cord lesion [70–72]. The understanding of the neuroplasticity potential of the lesioned spinal cord would favor the translation of spinal cord regeneration to clinical practice (http://www.clinicaltrial.gov, NCT00406016) [73, 74].

### 4.2. PEDF Treatment and Neuroplasticity Responses

Neurotrophic factors have been tested on spinal cord injury experimentally, especially those with actions on several aspects of neurorestoration, for instance, wound repair, neuronal trophism, and neuroplasticity [75–77]. PEDF has been pointed out as a potential candidate mainly in terms of motor recovery because the molecule is highly expressed and triggered trophic actions to motor neurons and because its receptor is concentrated in ventral motor region of the spinal cord [1, 2]. In fact, we described motor behavior improvements and neuroplasticity responses in the low thoracic lesioned rats after a PEDF injection in the epicenter of the injury. The rats that were submitted to the experimental procedures were accompanied behaviorally by means of the inclined plane test, which evaluates muscle strength and endurance parameters required to keep the animal in a static position when its plane is submitted to a progressive increase of the inclination angle [78, 79].

The possibility of PEDF injection in the epicenter of a spinal cord photothrombotic injury to trigger neuroplasticity events in the lumbar regions of the organ was evaluated by means of the responses of structural protein MAP-2. The regulation of that neuronal microtubule protein in the lesioned nervous system has been associated with dendritic branching and synaptic plasticity [80]. Western blot analysis revealed increases of MAP-2 levels in ventral regions of the lumbar spinal cord after the ischemic lesion applied cranially, remarkably in the rats treated with PEDF, indicating that the neurotrophic factor is able to stimulate neuronal plasticity in the regions of the spinal cord distant to the wound. Quantitative microdensitometric image analysis of MAP-2 immunoreactivity revealed, at cellular level, increases of MAP-2 immunoreactivity in the perikarya and neuropil of large motoneurons in the ventral horn of the lumbar region, caudal to ischemic injury site, of rats treated with PEDF. Image analysis is not able to separate MAP-2 positive dendrites from the cell bodies profiles. The stereological method however allowed us to show the neuroplasticity responses taking place in the dendritic process of MAP-2 positive neurons of the ventral horn of PEDF treated rats. These results are in agreement with previous publication which treated a traumatic spinal cord injury with another neurotrophic factor [19], thus revealing the potential of spinal cord plasticity to lead a higher behavioral recovery after organ injury.

### 4.3. Substrate for PEDF-Induced Neuroplasticity in the Lesioned Spinal Cord

Neuroplasticity is favored by a complex intracellular signaling that takes place in the rescued regions of the lesioned nervous tissue [6]. In fact, the unaltered levels of the antiapoptotic factor Bcl-2 [81–83] in lumbar ventral region indicate the absence of local neuronal death, favoring further neuronal plasticity in that region.

Neuroplasticity is a result of events specially addressed by the responsive neurons, their neighbor nonneuronal cells, and extracellular matrix molecules [84–86]. Regarding that, PEDF seems to be a promising candidate to spinal cord repair due to its actions on spinal cord neurons [2] and ability of signaling to the endothelial and glial cells, the main actors of neurorestorative events in nervous tissue [3, 87].

Remarkable PEDF actions include those that are inhibitory to angiogenesis and astrogliogenesis [87, 88], thus with substantial impact to extracellular matrix elements. Although neovascularization is required for neuronal fiber growth in regions close to injury acutely [89], to our knowledge, there is no publication referring to a long lasting possible interaction of vasculature regulation and neuronal plasticity in regions distant to lesion. Nevertheless, a recent publication correlated a local PEDF expression to opposite responses of nerve fibber growth and neovessels in the cornea treated with the neurotrophic factor FGF-2 [90], which is in agreement with our results on PEDF-induced downregulation of laminin, a marker of small blood vessels, in lumbar spinal cord neuroplasticity responsive region that is distant from the injury.

Inhibitory actions of PEDF to astrocytes may have not interfered with ability of reactive astrocytes of the lesioned spinal cord to secrete CSPG [91] in the neuroplasticity responsive region caudally. Inhibitory influence of extracellular matrix molecules, especially CSPG, to fiber growth close to the spinal cord injury site has been described [92, 93]; however, there is a lack of descriptions on the regulation of CSPG in the neuroplasticity responsive spinal cord regions. The decreased level of 70 kDa CSPG in the motor ventral region of the lumbar levels, far from the photothrombotic injury site, in animals treated or not with PEDF and at a chronic period after surgery (6 weeks) already emphasizes the ability of the lesioned spinal cord to last a permissive microenvironment for neuronal plasticity [94, 95]. All in all, PEDF actions to nonneuronal actors of neurorestorative events seem to favor neuronal plasticity in the lesioned spinal cord.

Additional feature of PEDF is its capability to modulate the expression of other neurotrophic factors [5], thus amplifying neurorestorative events triggered by specific trophic molecules in the lesioned nervous system [96–99].

There are actually several neurotrophic factors that play paracrine/autocrine actions on the spinal cord motor neurons [96]. Based on that, we evaluated gene expression of NT-3, GDNF, BDNF, and FGF-2 by means of RT-PCR in the neuroplasticity responsive lumbar motor region of the spinal cord injury rats.

We do not know whether photothrombotic ischemia in the low thoracic level of the rat spinal cord has triggered gene expression of studied neurotrophic factors in the neuroplasticity responsive lumbar region acutely; however, at the later week 6th time point, PEDF treatment modulated the differentially NT-3 and GDNF gene regulation. The PEDF-induced downregulation of NT-3 gene expression in the late period after injury might be related to a fine tuning of neuroplasticity responses in the cord [100, 101] by an action on the descending corticospinal motor fibers [102, 103]. Furthermore, such a regulation that was induced by PEDF treatment might have favored the expression of a neurotrophic factor with stronger actions to postsynaptic spinal cord motor neurons. That would be actually the case for GDNF [5, 76, 97, 104].

The absence of a late gene regulation of BDNF and FGF-2 might be explained for their ability to modulate early events after injury like neuronal rescue, progenitor cell proliferation, glial reactivity, and wound repair [14, 105].

### 4.4. Relevance of Ephrin System in Neuroplasticity Response Region of Lesioned Spinal Cord

The bidirectional signaling system provided by the Eph receptors and their ligands was recently mentioned as a key regulator of neuroplasticity in central nervous system [106]. Ephrin signaling plays important actions regarding axonal guidance and synaptogenesis during development [107, 108]. The role of Ephrin on the lesioned spinal cord has been the subject of recent investigation [29, 109]. The ephrin function is highlighted on neurorestorative events mainly because of its ability to prompt communication between neuronal and nonneuronal cells [110, 111].

We have analyzed at biochemical and cellular levels several members of the ephrin A and B families as well as the receptor subtypes of the Eph A and B families that could play a role in the lesioned spinal cord [112]. The EphA6 and EphB2 signals that were not shown by western blot and immunohistochemistry were evaluated at molecular levels by employing RT-PCR.

Upregulations of ephrins and their Eph receptors have been described in reactive astrocytes close to rat spinal cord injury [29, 31, 113]. The regulation of ephrin system close to spinal cord injury has been associated with its ability to interfere with cell activation related to wound repair [31] and also to inhibit neurite outgrowth and axonal regeneration close to a wound repair region [114, 115]. Remarkably, increases of EphA4 in reactive astrocytes close to a scar region of the lesioned spinal cord were correlated to inhibition of axonal regeneration [116, 117], event that gained importance after the description of the improvement of axonal regeneration and reduction of glial scar in the EphA4-deficient spinal cord injury mouse [113]. Furthermore, EphA4 antagonist was able to block retrograde axonal degeneration, to increase axonal regeneration, and to improve motor behavior in lesioned spinal cord rats [109, 116].

The present analysis demonstrated regulation of ephrin system in the neuroplasticity responsive lumbar motor region after a chronic spinal cord low thoracic injury, remarkably the ephrins A2, B1, and B3 and the receptor EphA4. The details of their contribution to neuronal plasticity in the lesioned spinal cord must be further evaluated. Nevertheless, changes in the levels of ephrin B system were correlated to neuropathic pain due to their ability to regulate neuronal excitability at spinal cord [118].

Important finding of the present study was the upregulation of ephrin B2 in the neuroplasticity responsive region of PEDF treated rats compared to nontreated lesioned animals, thus with a special relevance to motor recovery. In fact, the EphA4/ephrin B3 bidirectional signaling in developing spinal motor neurons seems to be involved in locomotion function [119–122]. Because ephrin B2 is specifically present in astrocytes of neuroplasticity responsive area, as demonstrated by the double immunofluorescence analyses, that ephrin signalling may contribute to well describe the ability of glial cells to promote neuronal plasticity [123]. That possibility has to be evaluated further in future investigations. All in all, it is likely that long term spinal cord injury might employ ephrin signaling for spontaneous motor recovery, event that could be amplified by neurotrophic factor-induced regulation of specific ephrin molecules, as it is the case of PEDF.

Finally, long term ephrin regulation in the neuroplasticity responsive region of the lesioned spinal cord does not seem to require the transcription factor RhoA. RhoA impairs neurite outgrowth, by means of growth cone collapse, an event that could be restricted to wound areas [124, 125] where upregulation of RhoA mRNA and protein last on reactive astrocytes of the lesion site of a cord injury [126–128]. Further analyses are required for a further understanding of the RhoA function in the neuroplasticity areas after a long period after injury.

## 5. Conclusion

PEDF injection in the epicenter of a low thoracic rat spinal cord photothrombotic ischemic injury improved motor behavior and triggered neuroplasticity responses in the motor regions of lumbar levels far from the lesion site. Changes in the expression of extracellular matrix protein, neurotrophic factors, and molecules of the ephrin system may have favored neuroplasticity.

## Figures and Tables

**Table 1:** Eph and ephrins antibodies employed in the western blot analysis.

Antibody	Description	Brand	Dilution
EphA1	Rabbit polyclonal	Santa Cruz	—
EphA2	Rabbit polyclonal	Santa Cruz	1/200
EphA3	Rabbit polyclonal	Abcam	1/1,000
EphA4	Rabbit polyclonal	Santa Cruz	1/1,000
EphA5	Rabbit polyclonal	Santa Cruz	1/1,000
EphA6	Rabbit polyclonal	Santa Cruz	—
EphA7	Rabbit polyclonal	Santa Cruz	1/400
EphA8	Rabbit polyclonal	Santa Cruz	—
EphA10	Rabbit polyclonal	Santa Cruz	—
EphB1	Rabbit polyclonal	Santa Cruz	1/100
EphB2	Goat polyclonal	Santa Cruz	—
EphB3	Rabbit polyclonal	Santa Cruz	—
EphB4	Rabbit polyclonal	Santa Cruz	1/1,000
EphB6	Rabbit polyclonal	Abcam	1/200
Ephrin-A1	Rabbit polyclonal	Abcam	1/100
Ephrin-A2	Rabbit polyclonal	Santa Cruz	1/100
Ephrin-A3	Rabbit polyclonal	Santa Cruz	1/100
Ephrin-A4	Rabbit polyclonal	Santa Cruz	1/100
Ephrin-A5	Rabbit polyclonal	Santa Cruz	1/300
Ephrin-B1	Rabbit polyclonal	Santa Cruz	1/100
Ephrin-B2	Goat polyclonal	Santa Cruz	1/100
Ephrin-B3	Goat polyclonal	Santa Cruz	1/100

Eph and ephrin antibodies and respective dilutions used in the western blot experiments. Antibodies pointed with no dilution showed no specific bands and molecules were not evaluated biochemically.

**Table 2:** Neurotrophic factors, Eph receptors, and GTPase levels.

	Gene	Sham	Saline	PEDF
NFs	NT-3	1.295 ± 0.121	0.849 ± 0.146	0.796 ± 0.114∗
	GDNF	1.298 ± 0.134	0.925 ± 0.122	1.794 ± 0.149^∗ ##^
	BDNF	1.069 ± 0.113	1.276 ± 0.256	1.126 ± 0.138
	FGF-2	1.208 ± 0.139	1.113 ± 0.067	1.117 ± 0.091

Ephs	EphA6	0.915 ± 0.070	0.944 ± 0.114	1.210 ± 0.179
	EphB2	0.935 ± 0.062	0.915 ± 0.062	1.008 ± 0.076

GTPase	RhoA	1.132 ± 0.115	1.083 ± 0.063	1.070 ± 0.070

Relative gene expression of the neurotrophic factors (NFs) NT-3, GDNF, BDNF, and FGF-2, the EphA6 and EphB2 receptors, and the RhoA in ventral region of the spinal cord of Sham, Saline, and PEDF groups. ∗*P* < 0.05 difference between PEDF and Sham groups and ^##^ *P* < 0.05 the difference between PEDF and Saline groups by means of two-way ANOVA followed by Bonferroni posttest. Means ± s.e.m., *n* = 6–8.
